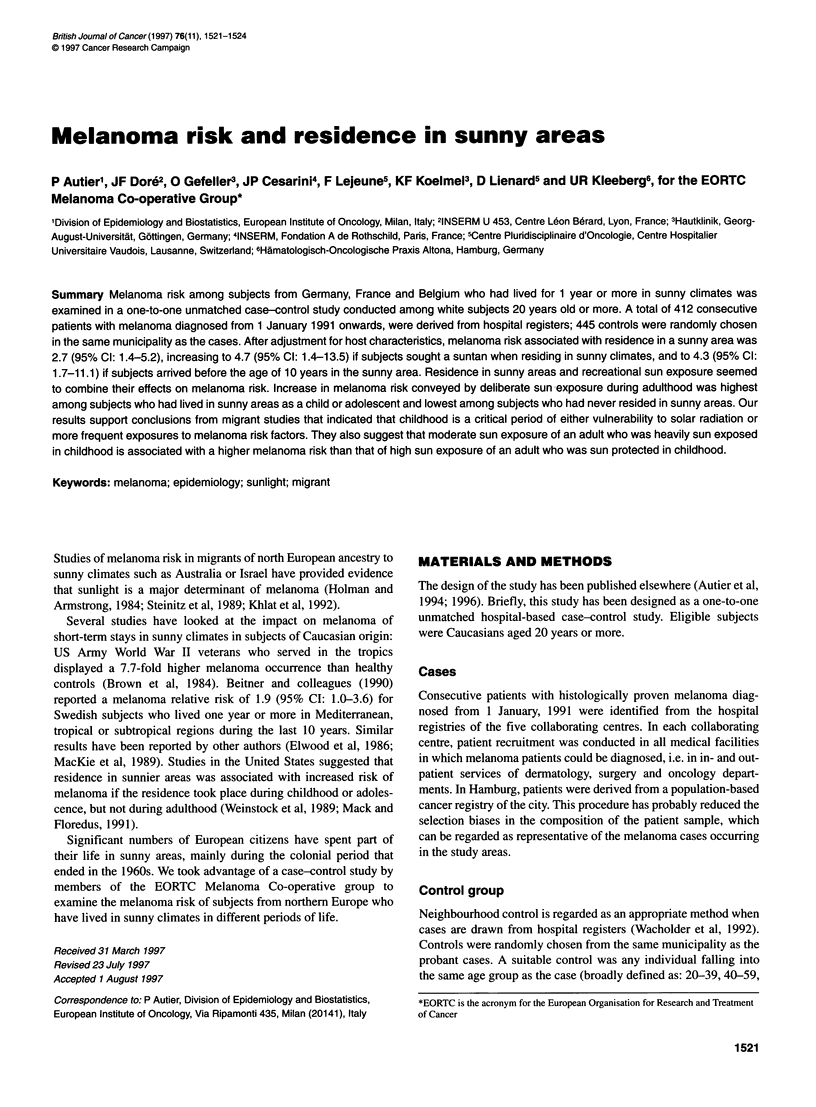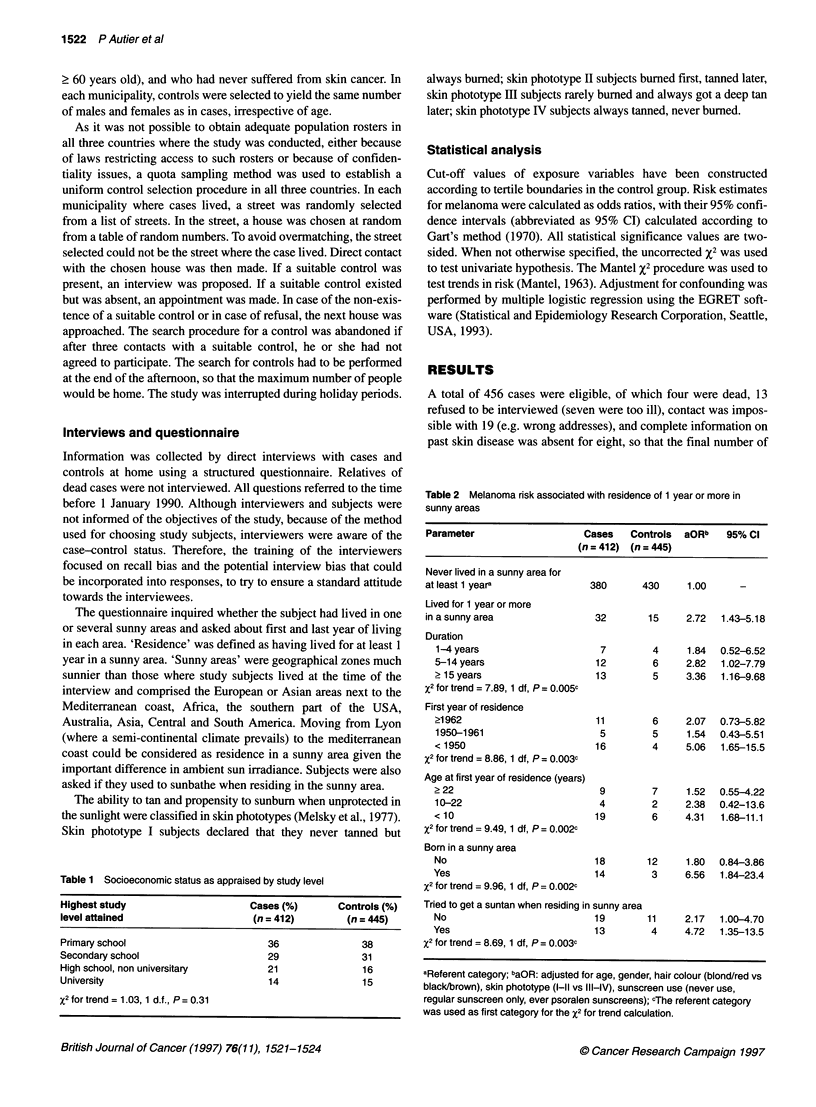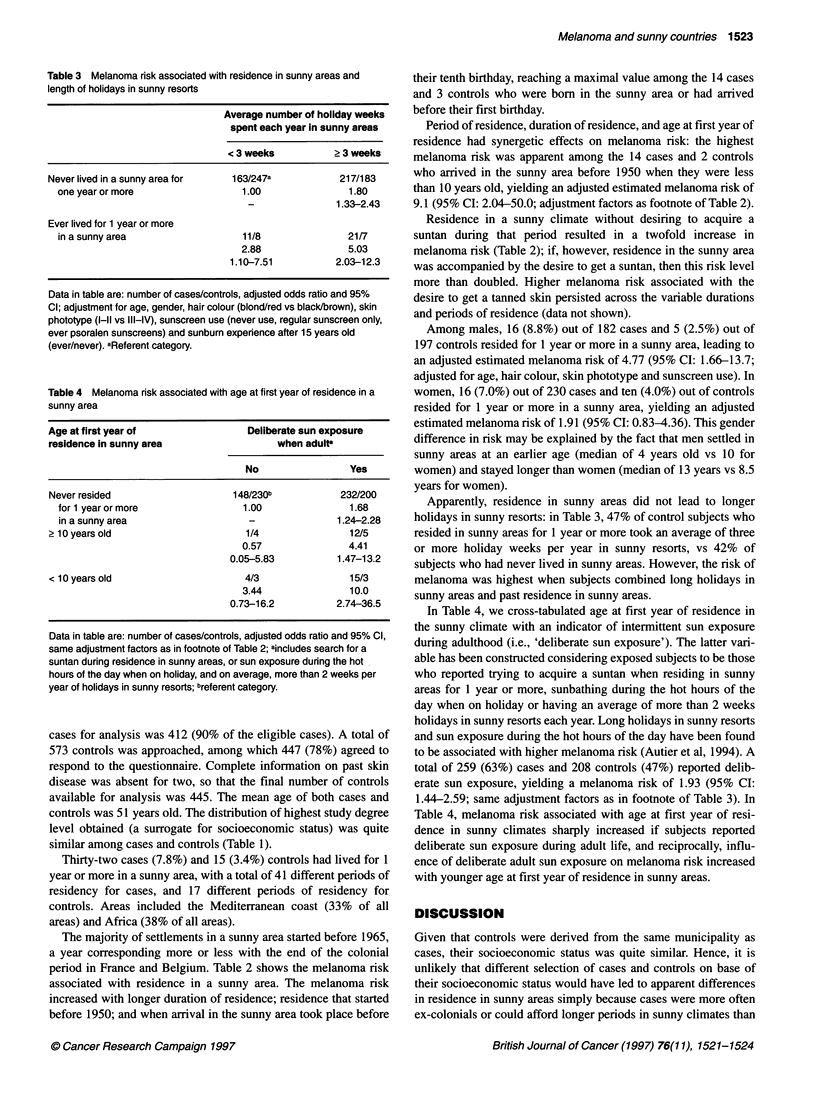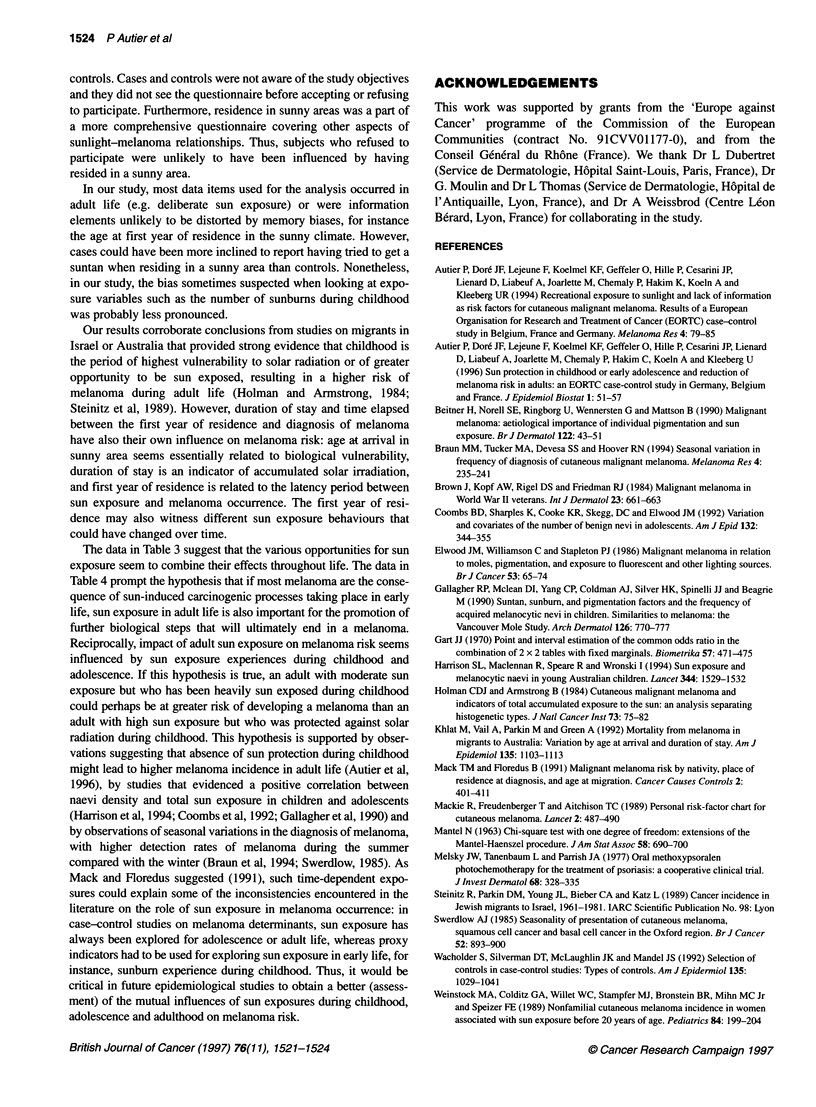# Melanoma risk and residence in sunny areas. EORTC Melanoma Co-operative Group. European Organization for Research and Treatment of Cancer.

**DOI:** 10.1038/bjc.1997.588

**Published:** 1997

**Authors:** P. Autier, J. F. DorÃ©, O. Gefeller, J. P. Cesarini, F. Lejeune, K. F. Koelmel, D. Lienard, U. R. Kleeberg

**Affiliations:** Division of Epidemiology and Biostatistics, European Institute of Oncology, Milan, Italy.

## Abstract

Melanoma risk among subjects from Germany, France and Belgium who had lived for 1 year or more in sunny climates was examined in a one-to-one unmatched case-control study conducted among white subjects 20 years old or more. A total of 412 consecutive patients with melanoma diagnosed from 1 January 1991 onwards, were derived from hospital registers; 445 controls were randomly chosen in the same municipality as the cases. After adjustment for host characteristics, melanoma risk associated with residence in a sunny area was 2.7 (95% CI: 1.4-5.2), increasing to 4.7 (95% CI: 1.4-13.5) if subjects sought a suntan when residing in sunny climates, and to 4.3 (95% CI: 1.7-11.1) if subjects arrived before the age of 10 years in the sunny area. Residence in sunny areas and recreational sun exposure seemed to combine their effects on melanoma risk. Increase in melanoma risk conveyed by deliberate sun exposure during adulthood was highest among subjects who had lived in sunny areas as a child or adolescent and lowest among subjects who had never resided in sunny areas. Our results support conclusions from migrant studies that indicated that childhood is a critical period of either vulnerability to solar radiation or more frequent exposures to melanoma risk factors. They also suggest that moderate sun exposure of an adult who was heavily sun exposed in childhood is associated with a higher melanoma risk than that of high sun exposure of an adult who was sun protected in childhood.


*British Journal of Cancer* (1997) **76**(11), 1521–1524
© 1997 Cancer Research Campaign

### Melanoma risk and residence in sunny areas

**P Autier[1], JF Doré[2], O Gefeller[3], JP Cesarini[4], F Lejeune[5], KF Koelmel[3], D Lienard[5] and UR Kleeberg[6], for the EORTC Melanoma Co-operative Group***

[1]Division of Epidemiology and Biostatistics, European Institute of Oncology, Milan, Italy; [2]INSERM U 453, Centre Léon Bérard, Lyon, France; [3]Hautklinik, Georg-August-Universität, Göttingen, Germany; [4]INSERM, Fondation A de Rothschild, Paris, France; [5]Centre Pluridisciplinaire d'Oncologie, Centre Hospitalier Universitaire Vaudois, Lausanne, Switzerland; [6]Hämatologisch-Oncologische Praxis Altona, Hamburg, Germany

**Summary** Melanoma risk among subjects from Germany, France and Belgium who had lived for 1 year or more in sunny climates was examined in a one-to-one unmatched case–control study conducted among white subjects 20 years old or more. A total of 412 consecutive patients with melanoma diagnosed from 1 January 1991 onwards, were derived from hospital registers; 445 controls were randomly chosen in the same municipality as the cases. After adjustment for host characteristics, melanoma risk associated with residence in a sunny area was 2.7 (95% CI: 1.4–5.2), increasing to 4.7 (95% CI: 1.4–13.5) if subjects sought a suntan when residing in sunny climates, and to 4.3 (95% CI: 1.7–11.1) if subjects arrived before the age of 10 years in the sunny area. Residence in sunny areas and recreational sun exposure seemed to combine their effects on melanoma risk. Increase in melanoma risk conveyed by deliberate sun exposure during adulthood was highest among subjects who had lived in sunny areas as a child or adolescent and lowest among subjects who had never resided in sunny areas. Our results support conclusions from migrant studies that indicated that childhood is a critical period of either vulnerability to solar radiation or more frequent exposures to melanoma risk factors. They also suggest that moderate sun exposure of an adult who was heavily sun exposed in childhood is associated with a higher melanoma risk than that of high sun exposure of an adult who was sun protected in childhood.

**Keywords:** melanoma; epidemiology; sunlight; migrant

Studies of melanoma risk in migrants of north European ancestry to sunny climates such as Australia or Israel have provided evidence that sunlight is a major determinant of melanoma (Holman and Armstrong, 1984; Steinitz et al, 1989; Khlat et al, 1992).

Several studies have looked at the impact on melanoma of short-term stays in sunny climates in subjects of Caucasian origin: US Army World War II veterans who served in the tropics displayed a 7.7-fold higher melanoma occurrence than healthy controls (Brown et al, 1984). Beitner and colleagues (1990) reported a melanoma relative risk of 1.9 (95% CI: 1.0–3.6) for Swedish subjects who lived one year or more in Mediterranean, tropical or subtropical regions during the last 10 years. Similar results have been reported by other authors (Elwood et al, 1986; MacKie et al, 1989). Studies in the United States suggested that residence in sunnier areas was associated with increased risk of melanoma if the residence took place during childhood or adolescence, but not during adulthood (Weinstock et al, 1989; Mack and Floredus, 1991).

Significant numbers of European citizens have spent part of their life in sunny areas, mainly during the colonial period that ended in the 1960s. We took advantage of a case–control study by members of the EORTC Melanoma Co-operative group to examine the melanoma risk of subjects from northern Europe who have lived in sunny climates in different periods of life.

*Received 31 March 1997*
*Revised 23 July 1997*
*Accepted 1 August 1997*

*Correspondence to:* P Autier, Division of Epidemiology and Biostatistics, European Institute of Oncology, Via Ripamonti 435, Milan (20141), Italy

### MATERIALS AND METHODS

The design of the study has been published elsewhere (Autier et al, 1994; 1996). Briefly, this study has been designed as a one-to-one unmatched hospital-based case–control study. Eligible subjects were Caucasians aged 20 years or more.

### Cases

Consecutive patients with histologically proven melanoma diagnosed from 1 January, 1991 were identified from the hospital registries of the five collaborating centres. In each collaborating centre, patient recruitment was conducted in all medical facilities in which melanoma patients could be diagnosed, i.e. in in- and out-patient services of dermatology, surgery and oncology departments. In Hamburg, patients were derived from a population-based cancer registry of the city. This procedure has probably reduced the selection biases in the composition of the patient sample, which can be regarded as representative of the melanoma cases occurring in the study areas.

### Control group

Neighbourhood control is regarded as an appropriate method when cases are drawn from hospital registers (Wacholder et al, 1992). Controls were randomly chosen from the same municipality as the probant cases. A suitable control was any individual falling into the same age group as the case (broadly defined as: 20–39, 40–59,

*EORTC is the acronym for the European Organisation for Research and Treatment of Cancer

≥ 60 years old), and who had never suffered from skin cancer. In each municipality, controls were selected to yield the same number of males and females as in cases, irrespective of age.

As it was not possible to obtain adequate population rosters in all three countries where the study was conducted, either because of laws restricting access to such rosters or because of confidentiality issues, a quota sampling method was used to establish a uniform control selection procedure in all three countries. In each municipality where cases lived, a street was randomly selected from a list of streets. In the street, a house was chosen at random from a table of random numbers. To avoid overmatching, the street selected could not be the street where the case lived. Direct contact with the chosen house was then made. If a suitable control was present, an interview was proposed. If a suitable control existed but was absent, an appointment was made. In case of the non-existence of a suitable control or in case of refusal, the next house was approached. The search procedure for a control was abandoned if after three contacts with a suitable control, he or she had not agreed to participate. The search for controls had to be performed at the end of the afternoon, so that the maximum number of people would be home. The study was interrupted during holiday periods.

### Interviews and questionnaire

Information was collected by direct interviews with cases and controls at home using a structured questionnaire. Relatives of dead cases were not interviewed. All questions referred to the time before 1 January 1990. Although interviewers and subjects were not informed of the objectives of the study, because of the method used for choosing study subjects, interviewers were aware of the case–control status. Therefore, the training of the interviewers focused on recall bias and the potential interview bias that could be incorporated into responses, to try to ensure a standard attitude towards the interviewees.

The questionnaire inquired whether the subject had lived in one or several sunny areas and asked about first and last year of living in each area. 'Residence' was defined as having lived for at least 1 year in a sunny area. 'Sunny areas' were geographical zones much sunnier than those where study subjects lived at the time of the interview and comprised the European or Asian areas next to the Mediterranean coast, Africa, the southern part of the USA, Australia, Asia, Central and South America. Moving from Lyon (where a semi-continental climate prevails) to the mediterranean coast could be considered as residence in a sunny area given the important difference in ambient sun irradiance. Subjects were also asked if they used to sunbathe when residing in the sunny area.

The ability to tan and propensity to sunburn when unprotected in the sunlight were classified in skin phototypes (Melsky et al., 1977). Skin phototype I subjects declared that they never tanned but always burned; skin phototype II subjects burned first, tanned later, skin phototype III subjects rarely burned and always got a deep tan later; skin phototype IV subjects always tanned, never burned.

**Table 1** Socioeconomic status as appraised by study level

| Highest study level attained | Cases (%) ($n$ = 412) | Controls (%) ($n$ = 445) |
|---|---|---|
| Primary school | 36 | 38 |
| Secondary school | 29 | 31 |
| High school, non universitary | 21 | 16 |
| University | 14 | 15 |
| $\chi^2$ for trend = 1.03, 1 d.f., $P$ = 0.31 | | |

### Statistical analysis

Cut-off values of exposure variables have been constructed according to tertile boundaries in the control group. Risk estimates for melanoma were calculated as odds ratios, with their 95% confidence intervals (abbreviated as 95% CI) calculated according to Gart's method (1970). All statistical significance values are two-sided. When not otherwise specified, the uncorrected $\chi^2$ was used to test univariate hypothesis. The Mantel $\chi^2$ procedure was used to test trends in risk (Mantel, 1963). Adjustment for confounding was performed by multiple logistic regression using the EGRET software (Statistical and Epidemiology Research Corporation, Seattle, USA, 1993).

### RESULTS

A total of 456 cases were eligible, of which four were dead, 13 refused to be interviewed (seven were too ill), contact was impossible with 19 (e.g. wrong addresses), and complete information on past skin disease was absent for eight, so that the final number of

**Table 2** Melanoma risk associated with residence of 1 year or more in sunny areas

| Parameter | Cases ($n$ = 412) | Controls ($n$ = 445) | aOR[b] | 95% CI |
|---|---|---|---|---|
| Never lived in a sunny area for at least 1 year[a] | 380 | 430 | 1.00 | – |
| Lived for 1 year or more in a sunny area | 32 | 15 | 2.72 | 1.43–5.18 |
| Duration | | | | |
| 1–4 years | 7 | 4 | 1.84 | 0.52–6.52 |
| 5–14 years | 12 | 6 | 2.82 | 1.02–7.79 |
| ≥ 15 years | 13 | 5 | 3.36 | 1.16–9.68 |
| $\chi^2$ for trend = 7.89, 1 df, $P$ = 0.005[c] | | | | |
| First year of residence | | | | |
| ≥1962 | 11 | 6 | 2.07 | 0.73–5.82 |
| 1950–1961 | 5 | 5 | 1.54 | 0.43–5.51 |
| < 1950 | 16 | 4 | 5.06 | 1.65–15.5 |
| $\chi^2$ for trend = 8.86, 1 df, $P$ = 0.003[c] | | | | |
| Age at first year of residence (years) | | | | |
| ≥ 22 | 9 | 7 | 1.52 | 0.55–4.22 |
| 10–22 | 4 | 2 | 2.38 | 0.42–13.6 |
| < 10 | 19 | 6 | 4.31 | 1.68–11.1 |
| $\chi^2$ for trend = 9.49, 1 df, $P$ = 0.002[c] | | | | |
| Born in a sunny area | | | | |
| No | 18 | 12 | 1.80 | 0.84–3.86 |
| Yes | 14 | 3 | 6.56 | 1.84–23.4 |
| $\chi^2$ for trend = 9.96, 1 df, $P$ = 0.002[c] | | | | |
| Tried to get a suntan when residing in sunny area | | | | |
| No | 19 | 11 | 2.17 | 1.00–4.70 |
| Yes | 13 | 4 | 4.72 | 1.35–13.5 |
| $\chi^2$ for trend = 8.69, 1 df, $P$ = 0.003[c] | | | | |

[a]Referent category; [b]aOR: adjusted for age, gender, hair colour (blond/red vs black/brown), skin phototype (I–II vs III–IV), sunscreen use (never use, regular sunscreen only, ever psoralen sunscreens); [c]The referent category was used as first category for the $\chi^2$ for trend calculation.

© Cancer Research Campaign 1997

**Table 3** Melanoma risk associated with residence in sunny areas and length of holidays in sunny resorts

| | Average number of holiday weeks spent each year in sunny areas | |
|---|---|---|
| | < 3 weeks | ≥ 3 weeks |
| Never lived in a sunny area for one year or more | 163/247[a] | 217/183 |
| | 1.00 | 1.80 |
| | – | 1.33–2.43 |
| Ever lived for 1 year or more in a sunny area | 11/8 | 21/7 |
| | 2.88 | 5.03 |
| | 1.10–7.51 | 2.03–12.3 |

Data in table are: number of cases/controls, adjusted odds ratio and 95% CI; adjustment for age, gender, hair colour (blond/red vs black/brown), skin phototype (I–II vs III–IV), sunscreen use (never use, regular sunscreen only, ever psoralen sunscreens) and sunburn experience after 15 years old (ever/never). [a]Referent category.

**Table 4** Melanoma risk associated with age at first year of residence in a sunny area

| Age at first year of residence in sunny area | Deliberate sun exposure when adult[a] | |
|---|---|---|
| | No | Yes |
| Never resided for 1 year or more in a sunny area | 148/230[b] | 232/200 |
| | 1.00 | 1.68 |
| | – | 1.24–2.28 |
| ≥ 10 years old | 1/4 | 12/5 |
| | 0.57 | 4.41 |
| | 0.05–5.83 | 1.47–13.2 |
| < 10 years old | 4/3 | 15/3 |
| | 3.44 | 10.0 |
| | 0.73–16.2 | 2.74–36.5 |

Data in table are: number of cases/controls, adjusted odds ratio and 95% CI, same adjustment factors as in footnote of Table 2; [a]includes search for a suntan during residence in sunny areas, or sun exposure during the hot hours of the day when on holiday, and on average, more than 2 weeks per year of holidays in sunny resorts; [b]referent category.

cases for analysis was 412 (90% of the eligible cases). A total of 573 controls was approached, among which 447 (78%) agreed to respond to the questionnaire. Complete information on past skin disease was absent for two, so that the final number of controls available for analysis was 445. The mean age of both cases and controls was 51 years old. The distribution of highest study degree level obtained (a surrogate for socioeconomic status) was quite similar among cases and controls (Table 1).

Thirty-two cases (7.8%) and 15 (3.4%) controls had lived for 1 year or more in a sunny area, with a total of 41 different periods of residency for cases, and 17 different periods of residency for controls. Areas included the Mediterranean coast (33% of all areas) and Africa (38% of all areas).

The majority of settlements in a sunny area started before 1965, a year corresponding more or less with the end of the colonial period in France and Belgium. Table 2 shows the melanoma risk associated with residence in a sunny area. The melanoma risk increased with longer duration of residence; residence that started before 1950; and when arrival in the sunny area took place before their tenth birthday, reaching a maximal value among the 14 cases and 3 controls who were born in the sunny area or had arrived before their first birthday.

Period of residence, duration of residence, and age at first year of residence had synergetic effects on melanoma risk: the highest melanoma risk was apparent among the 14 cases and 2 controls who arrived in the sunny area before 1950 when they were less than 10 years old, yielding an adjusted estimated melanoma risk of 9.1 (95% CI: 2.04–50.0; adjustment factors as footnote of Table 2).

Residence in a sunny climate without desiring to acquire a suntan during that period resulted in a twofold increase in melanoma risk (Table 2); if, however, residence in the sunny area was accompanied by the desire to get a suntan, then this risk level more than doubled. Higher melanoma risk associated with the desire to get a tanned skin persisted across the variable durations and periods of residence (data not shown).

Among males, 16 (8.8%) out of 182 cases and 5 (2.5%) out of 197 controls resided for 1 year or more in a sunny area, leading to an adjusted estimated melanoma risk of 4.77 (95% CI: 1.66–13.7; adjusted for age, hair colour, skin phototype and sunscreen use). In women, 16 (7.0%) out of 230 cases and ten (4.0%) out of controls resided for 1 year or more in a sunny area, yielding an adjusted estimated melanoma risk of 1.91 (95% CI: 0.83–4.36). This gender difference in risk may be explained by the fact that men settled in sunny areas at an earlier age (median of 4 years old vs 10 for women) and stayed longer than women (median of 13 years vs 8.5 years for women).

Apparently, residence in sunny areas did not lead to longer holidays in sunny resorts: in Table 3, 47% of control subjects who resided in sunny areas for 1 year or more took an average of three or more holiday weeks per year in sunny resorts, vs 42% of subjects who had never lived in sunny areas. However, the risk of melanoma was highest when subjects combined long holidays in sunny areas and past residence in sunny areas.

In Table 4, we cross-tabulated age at first year of residence in the sunny climate with an indicator of intermittent sun exposure during adulthood (i.e., 'deliberate sun exposure'). The latter variable has been constructed considering exposed subjects to be those who reported trying to acquire a suntan when residing in sunny areas for 1 year or more, sunbathing during the hot hours of the day when on holiday or having an average of more than 2 weeks holidays in sunny resorts each year. Long holidays in sunny resorts and sun exposure during the hot hours of the day have been found to be associated with higher melanoma risk (Autier et al, 1994). A total of 259 (63%) cases and 208 controls (47%) reported deliberate sun exposure, yielding a melanoma risk of 1.93 (95% CI: 1.44–2.59; same adjustment factors as in footnote of Table 3). In Table 4, melanoma risk associated with age at first year of residence in sunny climates sharply increased if subjects reported deliberate sun exposure during adult life, and reciprocally, influence of deliberate adult sun exposure on melanoma risk increased with younger age at first year of residence in sunny areas.

### DISCUSSION

Given that controls were derived from the same municipality as cases, their socioeconomic status was quite similar. Hence, it is unlikely that different selection of cases and controls on base of their socioeconomic status would have led to apparent differences in residence in sunny areas simply because cases were more often ex-colonials or could afford longer periods in sunny climates than

© Cancer Research Campaign 1997

controls. Cases and controls were not aware of the study objectives and they did not see the questionnaire before accepting or refusing to participate. Furthermore, residence in sunny areas was a part of a more comprehensive questionnaire covering other aspects of sunlight–melanoma relationships. Thus, subjects who refused to participate were unlikely to have been influenced by having resided in a sunny area.

In our study, most data items used for the analysis occurred in adult life (e.g. deliberate sun exposure) or were information elements unlikely to be distorted by memory biases, for instance the age at first year of residence in the sunny climate. However, cases could have been more inclined to report having tried to get a suntan when residing in a sunny area than controls. Nonetheless, in our study, the bias sometimes suspected when looking at exposure variables such as the number of sunburns during childhood was probably less pronounced.

Our results corroborate conclusions from studies on migrants in Israel or Australia that provided strong evidence that childhood is the period of highest vulnerability to solar radiation or of greater opportunity to be sun exposed, resulting in a higher risk of melanoma during adult life (Holman and Armstrong, 1984; Steinitz et al, 1989). However, duration of stay and time elapsed between the first year of residence and diagnosis of melanoma have also their own influence on melanoma risk: age at arrival in sunny area seems essentially related to biological vulnerability, duration of stay is an indicator of accumulated solar irradiation, and first year of residence is related to the latency period between sun exposure and melanoma occurrence. The first year of residence may also witness different sun exposure behaviours that could have changed over time.

The data in Table 3 suggest that the various opportunities for sun exposure seem to combine their effects throughout life. The data in Table 4 prompt the hypothesis that if most melanoma are the consequence of sun-induced carcinogenic processes taking place in early life, sun exposure in adult life is also important for the promotion of further biological steps that will ultimately end in a melanoma. Reciprocally, impact of adult sun exposure on melanoma risk seems influenced by sun exposure experiences during childhood and adolescence. If this hypothesis is true, an adult with moderate sun exposure but who has been heavily sun exposed during childhood could perhaps be at greater risk of developing a melanoma than an adult with high sun exposure but who was protected against solar radiation during childhood. This hypothesis is supported by observations suggesting that absence of sun protection during childhood might lead to higher melanoma incidence in adult life (Autier et al, 1996), by studies that evidenced a positive correlation between naevi density and total sun exposure in children and adolescents (Harrison et al, 1994; Coombs et al, 1992; Gallagher et al, 1990) and by observations of seasonal variations in the diagnosis of melanoma, with higher detection rates of melanoma during the summer compared with the winter (Braun et al, 1994; Swerdlow, 1985). As Mack and Floredus suggested (1991), such time-dependent exposures could explain some of the inconsistencies encountered in the literature on the role of sun exposure in melanoma occurrence: in case–control studies on melanoma determinants, sun exposure has always been explored for adolescence or adult life, whereas proxy indicators had to be used for exploring sun exposure in early life, for instance, sunburn experience during childhood. Thus, it would be critical in future epidemiological studies to obtain a better (assessment) of the mutual influences of sun exposures during childhood, adolescence and adulthood on melanoma risk.

### ACKNOWLEDGEMENTS

This work was supported by grants from the 'Europe against Cancer' programme of the Commission of the European Communities (contract No. 91CVV01177-0), and from the Conseil Général du Rhône (France). We thank Dr L Dubertret (Service de Dermatologie, Hôpital Saint-Louis, Paris, France), Dr G. Moulin and Dr L Thomas (Service de Dermatologie, Hôpital de l'Antiquaille, Lyon, France), and Dr A Weissbrod (Centre Léon Bérard, Lyon, France) for collaborating in the study.

### REFERENCES

Autier P, Doré JF, Lejeune F, Koelmel KF, Geffeler O, Hille P, Cesarini JP, Lienard D, Liabeuf A, Joarlette M, Chemaly P, Hakim K, Koeln A and Kleeberg UR (1994) Recreational exposure to sunlight and lack of information as risk factors for cutaneous malignant melanoma. Results of a European Organisation for Research and Treatment of Cancer (EORTC) case–control study in Belgium, France and Germany. *Melanoma Res* **4**: 79–85

Autier P, Doré JF, Lejeune F, Koelmel KF, Geffeler O, Hille P, Cesarini JP, Lienard D, Liabeuf A, Joarlette M, Chemaly P, Hakim C, Koeln A and Kleeberg U (1996) Sun protection in childhood or early adolescence and reduction of melanoma risk in adults: an EORTC case-control study in Germany, Belgium and France. *J Epidemiol Biostat* **1**: 51–57

Beitner H, Norell SE, Ringborg U, Wennersten G and Mattson B (1990) Malignant melanoma: aetiological importance of individual pigmentation and sun exposure. *Br J Dermatol* **122**: 43–51

Braun MM, Tucker MA, Devesa SS and Hoover RN (1994) Seasonal variation in frequency of diagnosis of cutaneous malignant melanoma. *Melanoma Res* **4**: 235–241

Brown J, Kopf AW, Rigel DS and Friedman RJ (1984) Malignant melanoma in World War II veterans. *Int J Dermatol* **23**: 661–663

Coombs BD, Sharples K, Cooke KR, Skegg, DC and Elwood JM (1992) Variation and covariates of the number of benign nevi in adolescents. *Am J Epid* **132**: 344–355

Elwood JM, Williamson C and Stapleton PJ (1986) Malignant melanoma in relation to moles, pigmentation, and exposure to fluorescent and other lighting sources. *Br J Cancer* **53**: 65–74

Gallagher RP, Mclean DI, Yang CP, Coldman AJ, Silver HK, Spinelli JJ and Beagrie M (1990) Suntan, sunburn, and pigmentation factors and the frequency of acquired melanocytic nevi in children. Similarities to melanoma: the Vancouver Mole Study. *Arch Dermatol* **126**: 770–777

Gart JJ (1970) Point and interval estimation of the common odds ratio in the combination of 2 × 2 tables with fixed marginals. *Biometrika* **57**: 471–475

Harrison SL, Maclennan R, Speare R and Wronski I (1994) Sun exposure and melanocytic naevi in young Australian children. *Lancet* **344**: 1529–1532

Holman CDJ and Armstrong B (1984) Cutaneous malignant melanoma and indicators of total accumulated exposure to the sun: an analysis separating histogenetic types. *J Natl Cancer Inst* **73**: 75–82

Khlat M, Vail A, Parkin M and Green A (1992) Mortality from melanoma in migrants to Australia: Variation by age at arrival and duration of stay. *Am J Epidemiol* **135**: 1103–1113

Mack TM and Floredus B (1991) Malignant melanoma risk by nativity, place of residence at diagnosis, and age at migration. *Cancer Causes Controls* **2**: 401–411

Mackie R, Freudenberger T and Aitchison TC (1989) Personal risk-factor chart for cutaneous melanoma. *Lancet* **2**: 487–490

Mantel N (1963) Chi-square test with one degree of freedom: extensions of the Mantel-Haenszel procedure. *J Am Stat Assoc* **58**: 690–700

Melsky JW, Tanenbaum L and Parrish JA (1977) Oral methoxypsoralen photochemotherapy for the treatment of psoriasis: a cooperative clinical trial. *J Invest Dermatol* **68**: 328–335

Steinitz R, Parkin DM, Young JL, Bieber CA and Katz L (1989) Cancer incidence in Jewish migrants to Israel, 1961–1981. IARC Scientific Publication No. 98: Lyon

Swerdlow AJ (1985) Seasonality of presentation of cutaneous melanoma, squamous cell cancer and basal cell cancer in the Oxford region. *Br J Cancer* **52**: 893–900

Wacholder S, Silverman DT, McLaughlin JK and Mandel JS (1992) Selection of controls in case-control studies: Types of controls. *Am J Epidermiol* **135**: 1029–1041

Weinstock MA, Colditz GA, Willet WC, Stampfer MJ, Bronstein BR, Mihn MC Jr and Speizer FE (1989) Nonfamilial cutaneous melanoma incidence in women associated with sun exposure before 20 years of age. *Pediatrics* **84**: 199–204

© Cancer Research Campaign 1997